# Randomized controlled trial of Understanding Social Situations versus problem-solving training in improving social function in people with psychosis

**DOI:** 10.1017/S1355617726102070

**Published:** 2026-06-19

**Authors:** Joanna M. Fiszdon, Kaicheng Wang, Daniel Fulford, Lori Parente, Alexis Nasse, Jimmy Choi, David L. Roberts

**Affiliations:** 1 Psychology, VA Connecticut Healthcare System, USA; 2 Psychiatry, Yale University, USA; 3 Yale Center for Analytical Sciences and Division of Biostatistics, Yale University, USA; 4 VA Connecticut Healthcare System, USA; 5 Psychological and Brain Sciences, Boston University, USA; 6 Department of Occupational Therapy and Rehabilitation Sciences, Boston University, USA; 7 Olin Neuropsychiatry Research Center, Hartford HealthCare, Hartford, USA; 8 Department of Psychiatry and Behavioral Sciences, The University of Texas Health Science Center at San Antonio, USA

**Keywords:** Ecological momentary assessment, problem-solving therapy, remote administration, schizophrenia, social cognition, Veteran

## Abstract

**Objectives:**

Poor social function in people with psychosis has been linked to impairments in social cognition and neurocognition, which in turn have been identified as promising treatment targets. We developed a novel social cognitive intervention, Understanding Social Situations (USS), that minimizes cognitive load by leveraging delivery methods common to cognitive rehabilitation. USS focuses on making good judgments about what others may be feeling and thinking, and how they might respond in various social scenarios. In the current trial, we evaluated the efficacy of USS versus an active control.

**Methods:**

103 Veterans with psychotic spectrum disorders were randomized to two months of USS training or a problem-solving training matched for nonspecific treatment factors. Comprehensive assessments included clinician-aided self-report social functioning, social skills performance, and pre/post/follow-up ecological momentary assessments about the extent and quality of social interactions.

**Results:**

Participants in USS had significant within but not between group improvements on a clinician-aided self-report measure of social function. Social skills performance did not change for either condition. Momentary assessments revealed no change in frequency of social contacts, but those in the USS condition showed within-group improvements on comfort level during current social interactions and anticipated positive appraisals of future interactions. Those randomized to problem-solving training had significantly greater improvements in depression at post-training.

**Conclusions:**

More rigorous trials are needed evaluating the impact of social cognitive interventions on real-world social functioning. It may be particularly important to link interventions to lived social experience and to assess the quality of social interactions.

## Statement of Research Significance

**Research Question(s) or Topic(s)**: Social cognition has been proposed as a proximal target for interventions aimed at improving social function in people with psychotic spectrum disorders. Critiques of existing trials include poor study quality and insufficient focus on generalization of effects to everyday functioning. We conducted a rigorous randomized trial evaluating a novel social cognitive training versus a modified problem-solving training. **Main Findings**: The social cognitive training was associated with small, within-group improvements on social functioning, comfort level during current social interactions, and anticipated appraisals of future interactions, but not frequency of social interactions. Problem-solving training was associated with significant improvements in depression. **Study Contributions**: In evaluating social cognitive interventions it may be particularly important to assess not just the quantity, but also the quality of social interactions. We also speculate that similarly to cognitive remediation, incorporating methods to generalize skills to everyday situations may be necessary to evidence larger effects.

## Introduction

Schizophrenia and other psychotic spectrum disorders (PSD) are characterized by significant impairments in neurocognition and social cognition, which are in turn associated with poor social function (Green, 2017). Social cognition, or how an individual perceives, processes, interprets and responds to social information (Green *et al*., 2008), has been proposed to mediate the relationship between neurocognition and functioning in PSD (Fett *et al*., 2011; Halverson *et al*., 2019). As such, social cognition may be a promising treatment target for interventions aimed at improving everyday social behaviors and social function in people with PSD (Green, 2017).

The literature on social cognitive interventions is growing, with consistently reported effects on social cognitive skills (Kurtz & Richardson, 2012; Tan *et al*., 2018), though there is less evidence for generalization to social functioning. In a 2020 network meta-analysis of 44 studies of social cognitive interventions with and without additional cognitive remediation therapy (Nijman *et al*., 2020), only 25 studies assessed social functioning. While significant effects were reported for social functioning, they were only present for broad-based treatments that trained multiple facets of social cognition. In the most recent meta-analysis, Yeo and colleagues (Yeo *et al*., 2022), evaluated 42 separate controlled trials of social cognitive interventions, 22 of which included some measures of functioning. Effects on psychosocial functioning were small and not significant. Concerns have also been raised about the methodological quality of social cognitive intervention validation trials, with only 10–15% of studies evaluated in two recent reviews (Grant *et al*., 2017; Nijman *et al*., 2020) considered to have met the minimum threshold for study quality. Majority of trials were assessed as at moderate to high risk of bias due to factors like lack of randomization, unblinded assessors, lack of fidelity assessments, treatment as usual as the comparator condition, and small sample sizes. Additional critiques of existing social cognitive intervention research include insufficient psychometric data on social cognitive measures, lack of information about durability of effects, no independent replication, and limited evidence of generalization to functional outcomes (Grant *et al*., 2017; Horan & Green, 2019; Kurtz *et al*., 2016; Nijman *et al*., 2020).

Our group developed a novel social cognitive intervention called Understanding Social Situations (USS). USS focuses on how to make good judgments about what people may be thinking or feeling in social situations and how they might respond in different social scenarios. The training is perhaps unique from other social cognitive interventions targeting higher-order social cognition in that it uses methods from (neuro)cognitive remediation to reduce the potential impact of cognitive impairments. In a single-arm, double-baseline trial with 38 individuals with PSD, the training was well tolerated, with high treatment satisfaction (Fiszdon *et al*., 2017). There were large improvements on mastery of content presented during the training. There were also significant effects on several social cognitive measures. Baseline neurocognitive impairment did not impact amount of improvement.

These promising results led us to the current evaluation of USS in a rigorous randomized controlled trial (RCT). We compared USS to an active problem-solving intervention that focuses on different strategies for handling everyday problems and stressors. Given the primary, clinically most meaningful treatment target of social function, along with oft-noted psychometric problems of many social cognitive measures (e.g., Pinkham *et al*., 2018; Ziermans *et al*., 2026), our main outcomes of interest were social functioning, evaluated using clinician-assisted self-report, performance-based assessments, and ecological momentary assessments (EMA). We hypothesized that those in the USS condition would improve more on these variables than those in the active control condition. Additionally, we examined the impact of training on a proximal measure of USS content knowledge, and explored potential effects of the interventions on symptom, cognitive, and general functioning measures.

## Methods

Study procedures were conducted in accordance with the Helsinki Declaration. Study was approved by the VA Connecticut Healthcare System institutional review board. All participants provided written informed consent. The study was registered in clinicaltrials.gov: NCT04557124. For details of study protocol, including comprehensive descriptions of the two interventions, please see (Fiszdon *et al*., 2024).

### Participants

Participants were recruited from a Veterans Affairs medical center. Eligibility criteria were: Veteran with a DSM-5 diagnosis of psychotic disorder (e.g. schizophrenia, schizoaffective disorder, delusional disorder, psychosis NOS, affective disorders with psychotic features), aged 18 or older, able to provide written informed consent, and fluent in English. Exclusion criteria were: prior exposure to the USS intervention or currently enrolled in another treatment study which might affect social functioning, presence of medical or neurological condition known to impair brain function (e.g., dementia, moderate or greater traumatic brain injury), hospitalizations or changes in medications in past 30 days, or meeting criteria for substance use disorder in the past 30 days. Recruitment began in December 2020, with last enrollment in March 2024.

### Design and procedure

This was a RCT examining the efficacy of USS social cognitive training. In order to provide a rigorous test of treatment efficacy and promote equipoise and participant blinding to study hypotheses, the comparator condition was an active intervention, a problem-solving training called Moving Forward (MF). The study was described to participants as evaluating two different treatments aimed at improving day-to-day interactions and quality of life. Both interventions were delivered individually and matched on duration, intensity, and therapist contact. Total duration of the active study intervention was 8–10 weeks. Training sessions were recorded for later fidelity ratings. Comprehensive assessments were conducted at baseline, post-training (2 months), and follow-up (4 months). An additional assessment of the primary social functioning outcome was conducted at treatment midpoint (4 weeks). Participants who missed assessment timeframes but did not withdraw were allowed to participate in subsequent testing timepoints. Participants were paid for assessments and training sessions. Randomization used a 1:1 ratio. Permuted block design with variable block size, stratified by baseline social functioning, was generated by the study statistician. Study therapist and assessors did not have access to the randomization scheme. Study assessors were masked to treatment allocation.

### Measures

In addition to baseline demographic, psychosocial, diagnostic (Structured Clinical Interview for DSM-5, First *et al*., 2015) and intelligence estimate (Wechsler Abbreviated Scale of Intelligence, 2-subtest estimate, Wechsler, 1999) information, repeated assessments capturing social functioning, cognition, and psychiatric symptoms were also conducted. For a portion of participants who enrolled during the Covid-19 pandemic, self-report and interviewer-based assessments were conducted virtually.

#### Social functioning primary outcome

The primary social functioning outcome was the Social Functioning Scale (SFS) (Birchwood *et al*., 1990). The SFS was specifically developed for and validated with people with psychosis, and is considered by experts to be one of the best-known measures of real-world social functioning (Leifker *et al*., 2011). It provides past-month ratings on seven subscales: social withdrawal, interpersonal functioning, prosocial activities, recreation, level of independence/competence, level of independence/performance, and employment. Raw scores from each subscale are converted to scaled scores with a mean of 100 and a standard deviation of 15, and subscale scores are averaged to obtain SFS total score (Barrowclough & Tarrier, 1997).

#### Social functioning secondary outcomes

There were two secondary measures of social functioning. The Social Skills Performance Assessment (SSPA) (Patterson *et al*., 2001) is a brief, structured role-play assessment of social skill ability. Role plays are rated based on the quality of the social interaction. Item scores for each role-play were averaged and then summed together for a single SSPA score, with higher scores reflecting better performance.

We also captured ecological momentary assessment (EMA) information about real-world social interactions using smartphone-delivered surveys. These types of surveys are particularly useful as they can provide information not only about the quantity, but also the perceived quality of social interactions in the context of daily life. Four surveys per day were administered over a period of 7 days. Participants had 1 hour to answer survey questions. For participants who did not complete any surveys in a given day, we offered to extend the EMA assessment period by one day. Surveys queried possible avoidance or withdrawal (whether participants were currently alone or with others and whether participants had recently communicated with others). An aggregate score was also created to index social appraisal of anticipated near-future interactions (how comfortable they think they will feel in future social interactions, how much criticism or rejection they expect, how likely do they think they are to feel suspicious of others in future social interactions, and how well do they think they will be able to understand other people’s perspectives in future interactions). Those who indicated that they had had recent social interactions were queried on how much they had wished they had been alone while interacting with others, and aggregate scores were created to index appraisals of recent interactions (how well the participants thought they communicated, how well they thought they understood what the other person was trying to tell them, how rejected or criticized by the other person they felt, and whether they thought the other person found them likeable), and comfort level in recent interactions (how much they enjoyed the interaction, how comfortable they felt in the interaction, how connected or close they felt to the other person in the interaction, how easy they found talking to the other person). All responses were keyed so higher scores indicated better social functioning.

#### Additional measures

To assess mastery of social cognitive content taught during the USS training, we administered the USS Knowledge test, a 22-item measure developed by our team that queries concepts and skills that are the focus of USS training (see Fiszdon *et al*., 2017). Test items are similar but not identical to training stimuli (e.g. participant is shown picture of a woman in a workout outfit, running, and asked whether it’s a fact or a guess that she likes to exercise; participant is asked whether it’s true or false that if they feel strongly that something is true then it definitely is true), and higher scores indicate greater mastery of USS training content.

Psychiatric symptoms were captured using the interviewer-rated Positive and Negative Syndrome Scale (PANSS) (Kay *et al*., 1987), which consists of Likert-type ratings of positive and negative symptoms associated with psychosis, as well as general psychopathology. Total scores range from 30 to 120, with higher scores indicating greater symptomatology. The interviewer-rated Quality of Life Scale (QoL) (Heinrichs *et al*., 1984) was used to capture overall functioning, with higher scores indicative of better overall functioning. ICC’s of study assessors, relative to gold standard study PI (JMF) ranged from 0.85 to 0.97. The self-reported Patient Health Questionnaire (PHQ-9) (Kroenke & Spitzer, 2002) was also used to capture symptoms of depression, with higher scores indicating greater depression.

Cognition was assessed using subtests from the Matrics Consensus Cognitive Battery (MCCB) (Nuechterlein & Green, 2006), which was designed specifically to evaluate cognitive change in treatment trials (Nuechterlein *et al*., 2008). Due to the Covid pandemic prevalent at study start, we omitted three subtests that required more intensive face-to-face contact: Spatial Span, Mazes, and the Brief Visual Memory Test. From the remaining 7 subtests, we created a MCCB social cognition score that consisted of performance on the Mayer-Salovey-Caruso Emotional Intelligence Test: Managing Emotions (MSCEIT-ME) (Brackett & Salovey, 2006), and a MCCB neurocognitive composite that averaged *t*-scores for the remaining 6 subtests.

#### Treatment engagement, fidelity, and acceptability

Engagement was assessed by examining the number of participants who completed both cycles of training and number of training sessions attended. Treatment fidelity was assessed using scales developed by the study team. Items included whether the therapist reviewed previous session activities and any prior homework assignments, whether the therapist provided modeling of training content, and whether the therapist adjusted training pace as needed and provided sufficient scaffolding and/or cueing so participant was able to complete exercises correctly. Also rated was overall quality of the delivered intervention, including therapeutic competence.

Participants were also asked to answer a brief, 4-point bidirectional (strongly disagree, disagree, agree, strongly agree) treatment acceptability survey. Items queried whether the training was easy to understand, how helpful and useful it was, and whether it helped participants better understand social situations and better understand other people.

### Study interventions

Both interventions were delivered individually, via telehealth or face-to-face delivery based on participant preference. Training content was delivered over 7–10, twice weekly, 45–60 minute sessions. To further consolidate any new learning, all participants were asked to go through two cycles of the training, making total intervention duration approximately 8–10 weeks and total number of sessions 14–20. The same study therapist delivered both interventions.

#### Understanding Social Situations (USS) social cognitive training

Training content for this intervention was mostly adapted from successful lab-based experimental interventions targeting theory of mind and attributional bias, with four modules providing computer-delivered exercises focused on distinguishing facts from guesses, evaluating how good or bad different guesses are based on how much evidence there is in support of them, using verbal mediation to more comprehensively process information relevant to making guesses about other peoples’ intentions, and attention bias modification exercises intended to induce a positive interpretive bias for ambiguous situations.

Since impairments in neurocognition have been suggested to limit skill acquisition (Grant *et al*., 2017; Kurtz & Richardson, 2012; Roberts & Velligan, 2012), techniques common to neurocognitive remediation were used throughout in order to lessen cognitive load. These techniques included modeling, breaking skills into subcomponents, scaffolding, hierarchical training, performance-based increases in task difficulty, and massed drill and practice. Each module contains multiple difficulty levels, and begins with basic information about a skill, and then proceeds to trainer-assisted drill and practice of exercises targeting that skill. For example, various photographs of social situations are shown and with assistance from the trainer so as to reduce errors, the participant is guided through making decisions about what features of the situation are facts versus guesses. As the training progresses, the distinction between facts and (good) guesses becomes less obvious (e.g. initially the participant may be asked whether it’s a fact or a guess that a person is wearing a watch, or whether a person that is looking at their watch is thinking about turtles, but with later content they may be asked whether the person looking at their watch is thinking about what time it is). At the end of each session homework is presented, which is reviewed at the beginning of the next session. Please see Fiszdon *et al*., 2024 for additional details.

#### Moving forward problem-solving training

Our active comparator training was a modification of “Moving Forward: Overcoming Life’s Challenges” (https://www.veterantraining.va.gov/movingforward) (MF) (Tenhula *et al*., 2014), a web-based problem-solving training focused on developing strategies to address common challenges including managing stress, relationship challenges, financial difficulties, adjustment issues, and other common problems.

### Statistical analysis

The study was powered to detect a clinically meaningful, moderate effect on the primary outcome measure, SFS. Based on a standard deviation of 13.1 (unpublished pilot data), we calculated that a sample size of 46 participants per group would be required to detect a 9-point difference (effect size *d* = 0.69) between the USS and MF at post-training, with 90% power and a two-sided significance level of 0.05.

Intention-to-treat analyses were conducted and included all randomized participants. Baseline characteristics were summarized as frequencies (percentages) and medians (interquartile ranges). Differences in treatment acceptability ratings between conditions were compared using the Wilcoxon rank-sum test. All analyses were performed using SAS version 9.4 (SAS Institute Inc., 2013), with statistical significance set at a two-tailed alpha <0.05.

To explore whether baseline MCCB neurocognition impacted amount of learning in USS (as indexed by improvement on the USS Knowledge test), a mixed-effects model was run among participants in the USS group. Repeated measures linear mixed-effects models were used to compare changes from baseline between the USS and MF arms in social functioning (SFS), social skill performance (SSPA), ecological momentary assessment (EMA) variables, USS Knowledge test, PANSS, QoL, PHQ-9, and the MCCB social cognition and neurocognitive composites. Score changes were calculated by subtracting baseline scores. All available measurements during the study period were included in the models. Mixed-effects models included fixed effects for categorical time, intervention group, the intervention-by-time interaction, baseline score, and assessment approach (in-person versus remote), along with a random intercept for participants. Estimated marginal means (least-squares means) were used to estimate changes from baseline at post-training (2 months) and follow-up (4 months), as well as between-group differences in change (difference-in-differences) between the USS and MF arms, using the LSMEANS and ESTIMATE statements in PROC MIXED. Subgroup analyses were also performed for SFS, SSPA and Knowledge test by sex and race. Additionally, an as-treated analysis was performed among participants who attended at least one training session.

## Results

### Sample characteristics

**Figure 1.**
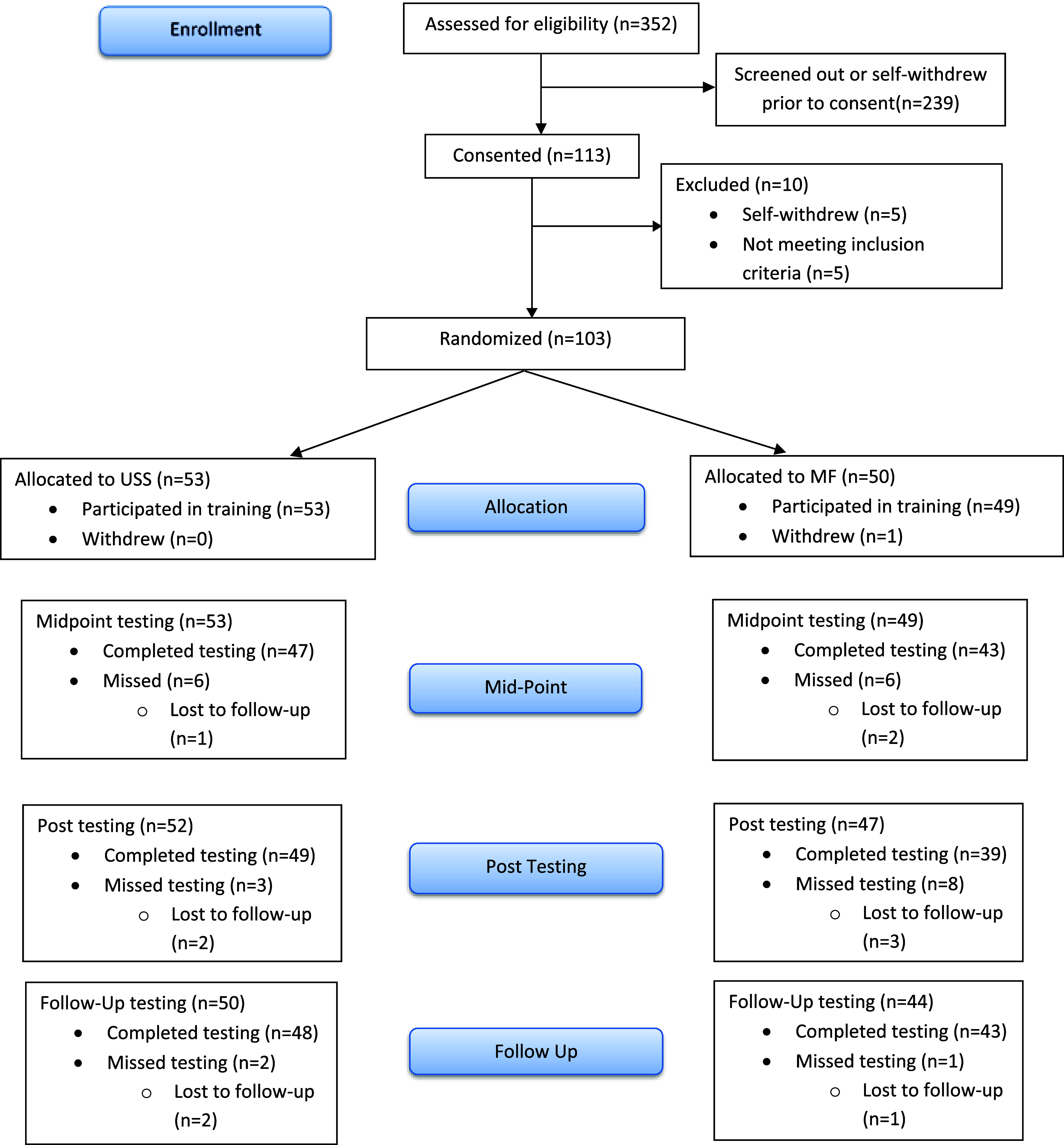
Figure 1 long description. CONSORT flow diagram.

A total of 113 participants signed informed consent and 103 were enrolled in this study, with 53 randomized to USS and 50 to MF (See CONSORT, Figure 1). Baseline characteristics are presented in Table 1. The median (IQR) age of all participants was 60.00 (42.00–66.00) years, with 13.59% women, 61.17% White, and 11.65% Hispanic or Latino. Vast majority (94 of 103) were diagnosed with a primary psychotic disorder, while remainder of the sample was split between Bipolar I disorder with psychotic features (*n* = 4) and Major Depressive Disorder with psychotic features (*n* = 5). Baseline demographics and clinical measures were comparable between USS and MF arms.

**Table 1.** Baseline characteristics of study participants Table 1 long description.

	MF (N = 50)	USS (N = 53)
**Age**, median (IQR)	61.00 (47.00–68.00)	60.00 (42.00–64.00)
**Sex**, female	8 (16.00%)	6 (11.32%)
**Race**		
White	28 (56.00%)	35 (66.04%)
Black or African American	22 (44.00%)	13 (24.53%)
More than one race	0 (0.00%)	2 (3.77%)
Native American	0 (0.00%)	3 (5.66%)
**Ethnicity**, Hispanic or Latino	5 (10.00%)	7 (13.21%)
**Marital status**		
Married/divorced/widowed	22 (44.00%)	29 (54.72%)
Never married	28 (56.00%)	24 (45.28%)
**Education**, yrs, median (IQR)	13.00 (12.00–14.00)	13.00 (12.00–15.00)
**Number of hospitalizations**, median (IQR)	3.50 (2.00–10.00)	3.50 (2.00–8.00)
**WASI IQ**, median (IQR)	99.00 (90.00–110.00)	100.00 (91.00–108.00)
**PANSS**, median (IQR)	48.50 (42.00–61.00)	51.00 (46.00–60.00)
**MCCB neurocognitive,** median (IQR)	37.00 (31.67–42.33)	37.00 (30.33–42.67)
**MCCB social cognition,** median (IQR)	39.00 (32.00–47.00)	42.00 (32.00–49.00)
**QoL**, median (IQRQ)	70.50 (62.00–90.00)	82.00 (66.00–90.00)
**PHQ-9**, median (IQR)	9.00 (3.50–13.00)	7.00 (4.00–14.00)
Missing	6	6
**SFS total**, median (IQR)	107.32 (99.50–111.57)	105.57 (98.79–111.07)
**SSPA**, median (IQR)	7.38 (6.90–8.03)	7.42 (7.13–8.40)
**USS Knowledge test**, median (IQR)	17.00 (16.00–20.00)	17.00 (16.00–19.00)

*Note*: WASI IQ = Wechsler abbreviated scale of intelligence, intelligence quotient; PANSS = positive and negative syndrome scale; MCCB = matrics consensus cognitive battery; QoL = quality of life scale; PHQ-9 = patient health questionnaire 9 item; SFS = social functioning scale; SSPA = social skills performance assessment.

Bold data cells indicate a statistically significant change (*p* < 0.05).

The median (IQR) SFS score was 105.57 (98.79–111.07) in the USS arm and 107.32 (99.50–111.57) in the MF arm, while the median (IQR) SSPA score was 7.42 (7.13–8.40) in the USS arm and 7.38 (6.90–8.03) in the MF arm.

Baseline neurocognitive performance did not have a significant impact on amount of learning on the USS Knowledge test – for participants in the USS condition, a one point increase in MCCB neurocognitive composite score was associated with 0.02 decrease in USS Knowledge test (*p* = 0.512). The first 23 participants who were enrolled during the Covid-19 pandemic, plus an additional 3 participants who were unable to come to the testing site, completed portions of the assessments remotely. All but two participants elected to receive the training via telehealth.

### Treatment engagement, fidelity, and acceptability

There was some variability in how quickly participants progressed through training content, with participants completing both rounds of training in between 14 and 20 sessions. Excluding 4 participants (1 in USS) who withdrew after randomization and did not receive any intervention, the number of sessions completed ranged from 4–20 for MF and 12–20 for USS. For USS, median [IQR] number of sessions completed was 20 [17–20], with 87% of the sample completing both training cycles, and an additional 9% completing at least one cycle. For MF, median [IQR] number of sessions completed was 17 [15–19], with 86% of the sample completing both training cycles, and an additional 8% completing at least 1 cycle.

Twenty-two participants (11 from each arm) were randomly selected for treatment fidelity ratings. For each participant, all available session recordings were rated. Fidelity to both interventions was very high: 96.52% for USS and 97.50% for MF.

Treatment acceptability was high, with mean item ratings in the agree-strongly agree range. Irrespective of randomization, training materials were rated as easy to understand (3.61/4), helpful (3.51/4), and as useful (3.47/4). Participants also indicated they thought the trainings would help them better understand social situations (3.47/4) and better understand other people (3.24/4). The two conditions were rated similarly on most items (*p* > 0.30), with the exception of whether participants thought the training was helpful, where there was a trend for the MF condition to score this item higher (MF = 3.59, USS = 3.44, *p* = 0.16), and whether participants thought the training would help them better understand other people, where there was a trend for participants in the USS condition to indicate higher agreement (MF = 3.11, USS = 3.35, *p* = 0.12). For the MF condition, participants particularly appreciated and related to video vignettes that featured actual service members describing their experiences. For the USS condition, participants commented on the training increasing their awareness, helping them pay attention to details, and helping them look at information more objectively.

### EMA compliance

Due to intermittent technical problems with survey provider, five participants were unable to complete surveys during one of the assessment timepoints. There were also three participants who declined to complete EMA surveys at baseline, and two who declined to complete them at post and at follow-up. At baseline, of 97 participants who were sent surveys (2716 surveys total), completion rate was 78.76%. At post training, 87 participants were sent surveys (2436 surveys total), and completion rate was 70.98%. At follow-up, 86 participants were sent surveys (2408 surveys total), and completion rate was 69.31%.

### Primary outcome

Least squares mean estimates of post–pre changes and differences between the USS and MF arms are presented in Table 2. At post-training (2 months), participants in the USS arm showed a 1.28-point improvement in SFS compared to a 0.19-point improvement in the MF arm. However, these changes, as well as the difference between the USS and MF arms, were not statistically significant. At follow-up (4 months), participants in the USS arm demonstrated a significant 1.56-point (95% CI 0.11–3.01) within-group improvement in SFS. However, the between-group difference compared to the MF arm remained non-significant. While the study was sufficiently powered for the anticipated moderate effect size (9-point difference, *d* = 0.69), the actual effect size was much smaller (1.09 point change; 95% CI −0.71–2.89; Cohen’s *d* using post-testing raw data = 0.28). Results are consistent with the at-treated analyses after excluding 4 participants who were randomized but withdrew before receiving any training sessions (Table 3).

**Table 2.** Least squares estimation of the difference-in-differences of SFS, SSPA, USS Knowledge test, PANSS, MCCB, QoL and PHQ-9 Table 2 long description.

	Changes from baseline		USS vs. MF	
	USS (95% CI)	MF (95% CI)	Diff (95% CI)	*P*
**Post training (2 months)**				
SFS, total	1.28 (−0.04, 2.59)	0.19 (−1.22, 1.60)	1.09 (−0.71, 2.89)	0.234
SSPA	0.01 (−0.21, 0.22)	−0.11 (−0.34, 0.12)	0.12 (−0.17, 0.41)	0.400
USS Knowledge test	**2.22 (1.72, 2.71)**	**0.84 (0.32, 1.36)**	**1.38 (0.71, 2.04)**	**<0.001**
PANSS	−1.74 (−4.16, 0.68)	−0.96 (−3.57, 1.64)	−0.78 (−4.05, 2.50)	0.639
MCCB neurocognitive	**2.78 (1.36, 4.20)**	1.45 (−0.08, 2.98)	1.33 (−0.60, 3.25)	0.174
MCCB social cognition	0.45 (−2.07, 2.96)	**5.67 (2.96, 8.39)**	**−5.23 (−8.64, −1.82)**	**0.003**
QoL	−1.67 (−4.54, 1.21)	0.38 (−2.69, 3.45)	−2.05 (−5.89, 1.80)	0.293
PHQ-9	0.39 (−1.18, 1.95)	**−1.94 (−3.53, −0.35)**	**2.33 (0.49, 4.16)**	**0.014**
**Follow-up (4 months)**				
SFS, total	**1.56 (0.11, 3.01)**	0.32 (−1.18, 1.81)	1.24 (−0.73, 3.22)	0.215
SSPA	0.00 (−0.21, 0.22)	−0.03 (−0.24, 0.18)	0.03 (−0.25, 0.31)	0.812
USS Knowledge test	**2.22 (1.68, 2.76)**	**1.24 (0.67, 1.80)**	**0.98 (0.24, 1.73)**	**0.010**
PANSS	−1.91 (−4.43, 0.61)	−1.25 (−3.85, 1.36)	−0.66 (−4.04, 2.71)	0.697
MCCB neurocognitive	**2.37 (0.83, 3.92)**	**2.55 (0.95, 4.16)**	−0.18 (−2.27, 1.91)	0.864
MCCB social cognition	1.21 (−1.57, 3.99)	**4.07 (1.18, 6.96)**	−2.86 (−6.64, 0.92)	0.137
QoL	−1.56 (−4.84, 1.71)	−1.54 (−4.90, 1.82)	−0.02 (−4.44, 4.39)	0.991
PHQ-9	−0.07 (−1.75, 1.62)	−1.51 (−3.16, 0.14)	1.44 (−0.58, 3.47)	0.161

*Note*: SFS = social functioning scale; SSPA = social skills performance assessment; PANSS = positive and negative syndrome scale MCCB = matrics consensus cognitive battery; QoL = quality of life scale; PHQ-9 = patient health questionnaire 9 item.

Bold data cells indicate a statistically significant change (*p* < 0.05).

**Table 3.** As-treated analysis among participants who attended at least one training session Table 3 long description.

	Changes from baseline		USS vs. MF	
	USS (95% CI)	MF (95% CI)	Diff (95% CI)	*P*
**Post training (2 months)**				
SFS, total	1.28 (−0.04, 2.59)	0.19 (−1.22, 1.60)	1.09 (−0.71, 2.89)	0.234
SSPA	0.01 (−0.21, 0.22)	−0.11 (−0.34, 0.12)	0.12 (−0.17, 0.41)	0.416
**Follow-up (4 months)**				
SFS, total	**1.56 (0.11, 3.01)**	0.32 (−1.18, 1.81)	1.24 (−0.73, 3.22)	0.215
SSPA	0.00 (−0.21, 0.22)	−0.03 (−0.24, 0.18)	0.03 (−0.25, 0.31)	0.812

Least squares estimation of the difference-in-differences from mixed-effects models included fixed effects for categorical time, intervention group, the intervention-by-time interaction, baseline score, and assessment approach (in-person versus remote), along with a random intercept for participants.

*Note*: SFS = social functioning scale; SSPA = social skills performance assessment.

Bold data cells indicate a statistically significant change (*p* < 0.05).

### Secondary and additional outcomes

For SSPA, no significant changes were observed in the USS arm at post-training or follow-up compared to baseline, and the differences between the USS and MF arms were not statistically significant. In contrast, participants in the USS arm showed significantly greater improvements on the USS Knowledge Test compared to the MF arm at both post-training and follow-up.

No statistically significant improvements in PANSS or QoL were observed in either the USS or MF arms at post-training or follow-up. However, significant within-group improvements in MCCB neurocognitive scores were observed in the USS arm at post-training and follow-up, as well as in the MF arm at follow-up. Participants in the MF arm also demonstrated significant improvements in MCCB social cognition index at post-training and follow-up, as well as in PHQ-9 scores at post-training. The improvements in MCCB social cognition and PHQ-9 scores were significantly greater in the MF arm compared to the USS arm.

There were 53 participants in the USS condition and 44 participants in the MF condition who participated in the EMA surveys (Table 4). All participants endorsed having communicated with others at least once during each assessment period (baseline, post-training, follow-up). Differences between USS vs. MF were not statistically significant in terms of the likelihood of being alone (OR [95%CI] 1.25 [0.55–2.83] at post-training; 2.04 [0.92–4.52] at follow-up), or having recently communicated with other people (OR [95%CI] 0.90 [0.39–2.06], as assessed at post-training; 0.86 [0.36–2.07] at follow-up). Participants in the USS condition had an increase in anticipated positive appraisals of future interactions as assessed at post-training (mean 0.10; 95% CI 0.01–0.19) and follow-up (mean 0.11; 95% CI 0.02–0.20) compared to baseline, and less discomfort in current social interactions at post-training (mean 0.13; 95%CI 0.01–0.24); however, these improvements were not statistically significant compared to the changes observed in the MF group.

**Table 4.** Least squares estimation of the difference-in-differences of EMA Table 4 long description.

	Post-training vs. Baseline			2-month f/u vs. Baseline		
	USS (95% CI)	MF (95% CI)	DiD (95% CI)	USS (95% CI)	MF (95% CI)	DiD (95% CI)
***Overall sample (n)***	*53*	*44*	–	*53*	*44*	–
Avoidance/withdrawal, alone, OR (95% CI)	1.28 (0.76, 2.18)	1.03 (0.55, 1.92)	1.25 (0.55, 2.83)	1.28 (0.77, 2.16)	0.63 (0.34, 1.16)	2.04 (0.92, 4.52)
Avoidance/withdrawal, communicated, OR (95% CI)	0.78 (0.46, 1.35)	0.87 (0.46, 1.64)	0.90 (0.39, 2.06)	1.56 (0.88, 2.77)	1.82 (0.94, 3.52)	0.86 (0.36, 2.07)
Anticipated appraisals of future interactions, mean (95% CI)	**0.12 (0.02, 0.21)**	0.09 (−0.02, 0.20)	0.03 (−0.11, 0.17)	**0.13 (0.03, 0.22)**	0.06 (−0.04, 0.16)	0.05 (−0.08, 0.19)
***For episodes including communication (n)***	*53*	*44*	–	*53*	*44*	–
Negative appraisals of current interactions, mean (95% CI)	0.08 (−0.02, 0.17)	0.04 (−0.07, 0.15)	0.04 (−0.11, 0.19)	0.08 (−0.02, 0.18)	0.06 (−0.06, 0.17)	0.02 (−0.13, 0.17)
Discomfort in current social interactions, mean (95% CI)	**0.13 (0.01, 0.24)**	0.05 (−0.08, 0.18)	0.08 (−0.10, 0.25)	0.07 (−0.05, 0.18)	0.05 (−0.08, 0.19)	0.02 (−0.16, 0.19)
Current withdrawal from social interactions, mean (95% CI)	0.10 (−0.10, 0.31)	−0.17 (−0.40, 0.06)	0.27 (−0.03, 0.58)	0.12 (−0.09, 0.33)	−0.13 (−0.35, 0.10)	0.24 (−0.07, 0.55)

Bold data cells indicate a statistically significant change (*p* < 0.05).

### Sex and race subgroup analyses

As shown in Table 5, sex and race did not significantly impact changes on SFS or SSPA. Male participants did show significantly greater improvements than female participants on the USS Knowledge Test at post-training, which no longer significantly differentiated the groups at follow-up.

**Table 5.** Subgroup analysis of SFS, SSPA and Knowledge test score changes, by sex and race Table 5 long description.

	Male (95% CI)	Female (95% CI)	White (95% CI)	Non-White (95% CI)
**Post testing**				
SFS, total	0.30 (−1.63, 2.23)	2.75 (−2.34, 7.83)	2.10 (−0.30, 4.50)	−1.28 (−4.04, 1.47)
SSPA	0.16 (−0.15, 0.48)	−0.39 (−1.26, 0.48)	0.06 (−0.31, 0.43)	0.23 (−0.28, 0.74)
USS Knowledge Test	**1.40 (0.67, 2.13)**	0.92 (−1.40, 3.25)	**1.21 (0.36, 2.06)**	**1.38 (0.21, 2.55)**
**2 months f/u**				
SFS, total	0.76 (−1.34, 2.85)	3.23 (−2.54, 8.99)	2.10 (−0.30, 4.50)	−0.33 (−3.72, 3.06)
SSPA	0.04 (−0.26, 0.34)	−0.12 (−1.13, 0.89)	−0.00 (−0.37, 0.37)	0.09 (−0.40, 0.57)
USS Knowledge Test	**1.06 (0.25, 1.87)**	0.41 (−2.00, 2.81)	0.85 (−0.06, 1.76)	0.92 (−0.39, 2.22)

*Note*: SFS = social functioning scale; SSPA = social skills performance assessment.

Bold data cells indicate a statistically significant change (*p* < 0.05).

## Discussion

This was a rigorous RCT evaluating a novel social cognitive intervention, USS, against an existing problem-solving training, MF, that had been modified to match USS on non-specific treatment factors like duration, intensity, and therapist contact. Both treatments were delivered with high fidelity and had high acceptability ratings and completion rates. The USS condition was associated with significantly greater improvements on a measure of USS content knowledge, both at post and at follow-up. Baseline neurocognition did not impact the amount of improvement on the USS Knowledge test, suggesting we were successful in making the training accessible to participants with varied levels of cognitive ability. Contrary to our hypothesis, participants in the USS condition did not evidence superior outcomes on our primary social cognitive measure, SFS, though a within-condition significant increase was observed at follow-up.

There were no significant within or between condition effects on a secondary measure of social skills performance (SSPA). Analyses of secondary social functioning EMA data revealed no significant changes in how likely participants were to be alone, but there were within-group improvements for USS condition on how comfortable participants reported being in current social interactions (at post-training only), and how positive they expected future interactions to be (at both assessment timepoints). The finding of anticipated greater positive affect in future interactions is encouraging given emerging literature on its relationship to social anhedonia, a particularly refractory symptom often associated with psychosis (Catalano *et al*., 2026). Our findings are also in line with results from another social cognitive intervention trial (Cella *et al*., 2023) that found social cognitive training enhanced the quality of social interactions without impacting the quantity of actual social behaviors, as well as prior research suggesting that for people with psychosis, while rates of daily interactions may be similar to non-psychiatric controls, that the quality of these interactions may be poorer and they may be more frequently characterized as negative (Abel *et al*., 2021; Mote & Fulford, 2020). It is also notable that compared to those in the MF condition, those in the USS condition tended to rate their training as more likely to help them better understand other people.

Additional exploratory analyses revealed a significantly greater improvement in depression for the MF condition, which is consistent with prior literature on the efficacy of problem-solving therapy (Simon *et al*., 2024). There were also significant within-group improvements on neurocognition in the USS condition at both assessment timepoints, and in MF condition at follow-up only. Somewhat unexpectedly, MF condition was also associated with a significant improvement on the MCCB social cognitive emotional intelligence measure, which was present at both post and follow-up, and was significantly greater than in the USS condition. Upon reflection, this finding may not be wholly surprising, as the emotional intelligence measure focused on judging the perceived efficacy of different responses in reaching desired outcomes, and learning how to manage problems is a key aspect of problem-solving therapy, while USS training is focused on what may be a prerequisite for managing problems, i.e. understanding the nature of other people’s experiences There were no effects for either condition on PANSS or QoL, and race and gender did not significantly impact changes on our primary social function measure nor our secondary measure of social skills performance.

While we did not find the hypothesized robust signal for differential efficacy of USS on SFS, our findings are nevertheless important as they contribute to the small literature on the impact of social cognitive interventions on social functioning outcomes and, to our knowledge, are only the second (Cella *et al*., 2023 being the first) to include EMA outcomes in the context of this type of intervention. Additionally, the effect size on our primary social functioning measure, SFS, was much smaller than the anticipated moderate–large effect used to determine our sample size. As such, more encouraging (though still smaller) effects may have been observed with a larger sample.

It is possible that our problem-solving training was too active a comparator. While not specifically training social cognition, the training nevertheless teaches how to manage different challenges, including interpersonal challenges. It is plausible that results of such training may impact social functioning, whether through changes in how one manages social relationships, or through the observed changes in depression, which in turn could impact social approach or social perceptions. In short, while the treatments focused on different processes, both may have had some impact on aspects of social functioning. For this reason, the within-group findings should not be disregarded. Nevertheless, absolute size of within-group changes on our primary outcome was still small.

We did not include social cognitive measures in our assessment battery (with the exception of the MSCEIT measure present in MCCB) due to our concerns about the psychometric properties of these measures and given that our main outcome of interest was social functioning. In retrospect, including such measures may have been helpful to at least allow us to more directly compare our findings with those of other social cognitive interventions, and especially the large proportion of trials that included social cognitive but not social functioning outcomes. Given the relatively targeted nature of the USS intervention and the plethora of factors that likely impact social functioning (Bowie *et al*., 2008; Leifker *et al*., 2009; Yager *et al*., 2006), it may also have been overly optimistic to expect moderate–large changes in more distal measures of social functioning.

Even though our findings only showed within-group social function improvements for the USS condition on select measures, it is noteworthy that they were observed across varied measures of social functioning, including both a clinician-assisted self-report measure as well as real-world EMA reports. Particularly meaningful are the improvements on EMA assessments of the quality of social interactions. In order to evidence larger improvements in real-world social functioning, USS may have benefitted from being administered within a broader rehabilitation program and/or from incorporating bridging groups or other methods to generalize skills learned to everyday interactions – factors considered by experts to be important components of effective cognition-focused interventions (Bowie *et al*., 2020; Wykes *et al*., 2011).

Several unique characteristics of our study also warrant discussion. Our sample was all Veteran, with average age of 60 and baseline SFS score a few points above norms for this population. As such, it may not be representative of samples recruited for other social cognitive studies, and our findings may not be generalizable to younger samples or those with greater social function impairments. Older age and the associated longer total illness duration and more entrenched behavioral patterns may have impacted participant’s interest or ability to change how they perceive social interactions or how they view and interact with others. On the flip side, and as has been observed in the cognitive remediation literature (Reser *et al*., 2019), it is also possible that greater levels of improvement may have been observed if baseline level of the treatment target was lower.

While social cognition has been identified as an important contributor to social functioning and a potential treatment target, much more work is needed. Specifically needed are large, rigorous intervention trials that have long follow-up periods, and that examine real-world social functioning effects. As we observed and as has been suggested by others, particularly relevant may be assessing the impact of these relatively new interventions on not just the quantity, but also the quality of social interactions, as well as enhancing methods to link social cognitive interventions to participants’ lived social experience.

## Figure 1. Long description

The flowchart begins with the enrollment phase, where 352 participants are assessed for eligibility. 239 participants are screened out or self-withdraw prior to consent, leaving 113 participants who consent. 10 participants are excluded due to self-withdrawal or not meeting inclusion criteria, resulting in 103 participants being randomized. The allocation phase splits the participants into two groups: 53 allocated to USS and 50 allocated to MF. All 53 USS participants and 49 MF participants undergo training, with one MF participant withdrawing. At the midpoint testing phase, 47 USS participants and 43 MF participants complete testing, while 6 USS and 6 MF participants miss testing, with some lost to follow-up. In the post-testing phase, 49 USS participants and 39 MF participants complete testing, with 3 USS and 8 MF participants missing testing, and some lost to follow-up. The follow-up testing phase shows 48 USS participants and 43 MF participants completing testing, with 2 USS and 1 MF participant missing testing, and some lost to follow-up.

Navigate back to Figure 1..

## Table 1. Long description

The table compares baseline characteristics of study participants in MF and USS groups. It includes data on age, sex, race, ethnicity, marital status, education, number of hospitalizations, WASI IQ, PANSS, MCCB neurocognitive and social cognition scores, quality of life, PHQ-9, SFS total, SSPA, and USS Knowledge test. The table has 16 rows and 10 columns, with headers for each characteristic and columns for MF and USS groups. Notable trends include similar median ages, education levels, and IQ scores between the groups, with slight variations in race, ethnicity, and marital status distributions.

Navigate back to Table 1..

## Table 2. Long description

The table presents least squares estimation of the difference-in-differences for various metrics between USS and MF groups. It includes data for post-training (2 months) and follow-up (4 months). The table has 14 rows and 6 columns, with headers for Changes from baseline (USS and MF with 95% CI), USS vs. MF (Diff with 95% CI), and P value. Key metrics include SFS, SSPA, USS Knowledge test, PANSS, MCCB neurocognitive and social cognition, QoL, and PHQ-9. Notable trends include significant improvements in USS Knowledge test and PHQ-9 at post-training, and SFS at follow-up within the USS group. The between-group differences are generally non-significant, except for MCCB social cognition at post-training and USS Knowledge test at follow-up.

Navigate back to Table 2..

## Table 3. Long description

The table presents least squares mean estimates of changes from baseline and differences between USS and MF arms. It includes data for post-training (2 months) and follow-up (4 months). The table has four rows and three columns. The columns are labeled Changes from baseline, USS (95% CI), MF (95% CI), Diff (95% CI), and P. The rows are labeled Post training (2 months) and Follow-up (4 months), with sub-rows for SFS total and SSPA total. At post-training, USS shows a 1.28-point improvement in SFS compared to a 0.19-point improvement in MF, with a non-significant difference. At follow-up, USS shows a significant 1.56-point improvement in SFS, but the difference compared to MF remains non-significant. The table also includes confidence intervals and p-values for each comparison.

Navigate back to Table 3..

## Table 4. Long description

The table presents a comparison of least squares estimation of difference-in-differences of EMA across various metrics. It includes data for post-training versus baseline and 2-month follow-up versus baseline. The table has 11 rows and 7 columns, with column headers such as Overall sample, Avoidance/withdrawal alone, Avoidance/withdrawal communicated, Anticipated appraisals of future interactions, and more. Each row provides data for USS, MF, and DiD with their respective confidence intervals. Notable trends include variations in anticipated appraisals of future interactions and discomfort in current social interactions across different time points.

Navigate back to Table 4..

## Table 5. Long description

The table presents an analysis of SFS, SSPA, and Knowledge test score changes by sex and race. It includes data for post-testing and two-month follow-up periods. The table has four rows and five columns, with column headers for Male (95% CI), Female (95% CI), White (95% CI), and Non-White (95% CI). Row labels include SFS total, SSPA, and USS Knowledge Test for both post-testing and two-month follow-up. Notable trends include higher SFS total scores for females and whites in post-testing, and significant changes in USS Knowledge Test scores across all groups. The data cells indicate confidence intervals, with bold cells highlighting statistically significant changes (p less than 0.05).

Navigate back to Table 5..
